# FTO and ADRB2 Genetic Polymorphisms Are Risk Factors for Earlier Excessive Gestational Weight Gain in Pregnant Women with Pregestational Diabetes Mellitus: Results of a Randomized Nutrigenetic Trial

**DOI:** 10.3390/nu14051050

**Published:** 2022-03-01

**Authors:** Karina dos Santos, Eliane Lopes Rosado, Ana Carolina Proença da Fonseca, Gabriella Pinto Belfort, Letícia Barbosa Gabriel da Silva, Marcelo Ribeiro-Alves, Verônica Marques Zembrzuski, J. Alfredo Martínez, Cláudia Saunders

**Affiliations:** 1 Programa de Pós-Graduação em Nutrição, Instituto de Nutrição Josué de Castro, Universidade Federal do Rio de Janeiro, Avenida Carlos Chagas Filho, 373-Bloco J 2° andar, Cidade Universitária, Rio de Janeiro 21941-902, Brazil; karsantos@gmail.com (K.d.S.); elianerosado@nutricao.ufrj.br (E.L.R.); belfortgabriella@hotmail.com (G.P.B.); leticiabgs.nut04@gmail.com (L.B.G.d.S.); 2 Laboratório de Genética Humana, Instituto Oswaldo Cruz, Fundação Oswaldo Cruz, Pavilhão Leônidas Deane, Avenida Brasil 4365, Rio de Janeiro 21040-360, Brazil; ana_carol_pf@hotmail.com (A.C.P.d.F.); vezembrzuski@gmail.com (V.M.Z.); 3 Instituto Nacional de Infectologia Evandro Chagas, Fundação Oswaldo Cruz, Avenida Brasil 4365, Rio de Janeiro 21040-360, Brazil; mribalves@gmail.com; 4 Precision Nutrition and Cardiometabolic Health Program, IMDEA Food Institute, Crta. de Canto Blanco, n 8, E-28049 Madrid, Spain; jalfredo.martinez@imdea.org

**Keywords:** gestational weight gain, ADRB2, FTO, DASH diet, diabetes mellitus, nutrigenetics

## Abstract

Excessive gestational weight gain (GWG) is associated with increased risk of maternal and neonatal complications. We investigated obesity-related polymorphisms in the FTO gene (rs9939609, rs17817449) and ADRB2 (rs1042713, rs1042714) as candidate risk factors concerning excessive GWG in pregnant women with pregestational diabetes. This nutrigenetic trial, conducted in Brazil, randomly assigned 70 pregnant women to one of the groups: traditional diet (*n* = 41) or DASH diet (*n* = 29). Excessive GWG was the total weight gain above the upper limit of the recommendation, according to the Institute of Medicine guidelines. Genotyping was performed using real-time PCR. Time-to-event analysis was performed to investigate risk factors for progression to excessive GWG. Regardless the type of diet, AT carriers of rs9939609 (FTO) and AA carriers of rs1042713 (ADRB2) had higher risk of earlier exceeding GWG compared to TT (aHR 2.44; CI 95% 1.03–5.78; *p* = 0.04) and GG (aHR 3.91; CI 95% 1.12–13.70; *p* = 0.03) genotypes, respectively, as the AG carriers for FTO haplotype rs9939609:rs17817449 compared to TT carriers (aHR 1.79; CI 95% 1.04–3.06; *p* = 0.02).

## 1. Introduction

Excessive gestational weight gain (GWG) affects half of pregnancies worldwide [1] and nearly 40% of pregnancies in Brazil [2]. In women with pregestational diabetes mellitus (DM), excessive GWG is associated with a higher risk of preterm delivery, cesarean section, large-for-gestational-age newborn, macrosomia, neonatal distress, and neonatal malformations [3,4]. In addition to increasing the immediate risk of perinatal complications, excessive GWG is associated with short- and long-term metabolic consequences for mothers and children and probably plays a key role in the metabolic programming of chronic diseases in the offspring [5].

The GWG recommendations [6,7] are based on the pre-pregnancy body mass index (BMI), but genetics, dietetics, and environmental factors appear to be involved in significant interindividual variation in weight gain during pregnancy [8,9]. The fat mass and obesity-associated (FTO) gene is located on chromosome 16, and common polymorphisms in the first intron are strongly associated with obesity. FTO encodes a protein with demethylase function and is highly expressed in the hypothalamus, particularly in the arcuate nucleus, suggesting that this gene plays an essential role in energy balance and body weight control [10].

The polymorphisms rs9939609 (T/A) and rs17817449 (T/G) in FTO are associated with body weight, BMI, and extreme obesity in the Brazilian population [11]. The A allele of the polymorphism rs9939609 is associated with higher GWG in North America [12,13] and Spanish women [14] but not with excessive GWG in Brazilian women [15]. Despite the association of rs17817449 (GG) with higher maternal BMI in pregnant Iraqi women [16], its relationship with GWG has not yet been explored in the literature.

The adrenoceptor beta 2 (ADRB2) gene is located on chromosome 5, and some polymorphisms in this gene have been consistently associated with a predisposition to obesity due to its expression in adipose tissue and its role in lipolysis and energy balance [17]. Two common polymorphisms in ADRB2 are the most studied, rs1042713 (G/A) and rs1042714 (C/G), although the results are quite divergent, with only rs1042714 (CG/GG) associated with obesity in a meta-analysis [18]. The relationship between ADRB2 polymorphisms and GWG has not yet been explored.

Interventions that focus on a healthy diet have been found to be effective in optimizing GWG [5] and should start as early as possible, even during the periconceptional period [19]. The adherence to an “Western” dietary pattern—characterized by unhealthy and energy-dense foods with high intake of red meat, fries, dipping sauces, salty snacks, and alcoholic drinks—was associated with increased GWG, especially among obese women, in a cohort of women from Southern Europe [19].

Pregnant women with DM especially benefit from nutritional guidance to prevent excessive GWG and related adverse outcomes, which are more common in high-risk pregnancies [20]. The Dietary Approach to Stop Hypertension (DASH) diet encourages the consumption of fruits, vegetables, fat-free/low-fat dairy, whole grains, nuts, and legumes as well as limits the intake of saturated fat, cholesterol, refined sugar, sodium, red, and processed meats [21]. The DASH diet was originally proposed to control hypertension; however, in recent years, it has been recommended for pregnant women with DM, obesity, and hypertension to reduce the risk of obstetric and perinatal complications [22,23].

However, the effects of diet on body physiology vary greatly among individuals, which may be partially explained by nutrigenetic interactions. The association between FTO and ADRB2 genetic polymorphisms and obesity phenotypes appears to be modified by diet composition [24,25,26]. Personalized nutrition may benefit individuals genetically predisposed to have a higher BMI using a dietary pattern that minimizes risk [27]. Studies are needed to elucidate the association between genetic variants, diet, and GWG, especially for high-risk pregnancies, such as in mothers with pregestational DM.

The aim of this study was to investigate the FTO genetic polymorphisms (rs9939609, rs17817449) and the ADRB2 genetic polymorphisms (rs1042713, rs1042714) as candidate genetic risk factors for excessive GWG in pregnant women with pregestational DM using two different types of diets, a traditional diet and the DASH diet, as well as to ascertain nutrigenetic interactions associated to the genetic make-up.

## 2. Materials and Methods

### 2.1. Subjects

The participants were pregnant women who were enrolled in the DASDIA (DASh diet for pregnant women with DIAbetes) randomized controlled clinical trial carried out at the Maternity School of the Federal University of Rio de Janeiro, Rio de Janeiro, Brazil, with the aim of evaluating the effect of the DASH diet on perinatal outcomes in pregnant women with pregestational DM (2016–2020, Brazilian Clinical Trials Registry RBR-4tbgv6). Eighty-seven pregnant women participated in the DASDIA trial, of whom 70 were included in the present study because valid data were available for performing the nutrigenetic analyses.

The participants were pregnant women with pregestational DM; 18 years or older; less than 28 weeks pregnant at the time of inclusion in the study; single fetus; no alcohol, tobacco, or drug use; no sexually transmitted disease (e.g., syphilis, genital herpes, HPV); no psychiatric diseases (e.g., anxiety, depression, eating disorders); and no DM complications (e.g., diabetic nephropathy or retinopathy). Pregnant women with treated and controlled hypothyroidism (TSH 0.1–2.5 mUI/L in the first trimester or 0.3–3.0 mUI/L in the second trimester, using levothyroxine) or chronic hypertension (systolic blood pressure <160 mmHg and diastolic blood pressure <110, using methyldopa, without SHG) were included. The eligible participants were randomly assigned to one of two parallel study groups, traditional diet or DASH diet, using a computer-generated list of random numbers prepared by the head investigator (even numbers to DASH diet group and odd numbers to traditional diet group). The participants were blinded to allocation.

The research was approved by the Ethics Committee of the Maternity School of Federal University of Rio de Janeiro (CAAE–46913115.0.0000.5275; July/2015).

### 2.2. Pregestational Diabetes Diagnosis and Treatment

Women with type 1 or 2 DM were included in this study, with a pre-pregnancy diagnosis or diagnosed during pregnancy, after presenting with a fasting glucose level ≥126 mg/dL [20]. Women with gestational DM were excluded from the study. Following the institutional protocol, all participants were treated with insulin therapy, which was prescribed by physicians according to individual needs.

### 2.3. Diet Groups and Nutritional Guidance

The participants were randomly assigned to one of two groups of nutritional guidance, a traditional diet or the DASH diet. Women in both groups received individual nutritional guidance from a registered dietitian from the date of inclusion in the study until the last prenatal appointment in six scheduled visits. The time of intervention was defined as the time between inclusion in the study and childbirth.

Both traditional and DASH diets were designed to contain 45–55% carbohydrates, 15–20% protein, and 25–30% total fat. However, the DASH diet was richer in fruits, vegetables, whole grains, and low-fat dairy products and included a serving of nuts per day. The original North American version of the DASH diet was translated and adapted for the DASDIA trial, considering the characteristics of Brazilian pregnant women with DM, as detailed elsewhere [28]. The traditional diet was a healthy diet currently prescribed for all pregnant women with DM-attending prenatal care at the maternity hospital.

The main differences in the composition of the traditional and DASH diets based on the 2100 kcal meal plan are provided in Table 1.

The daily energy intake was calculated individually in order to formulate recommendations according to age, physical activity, pre-pregnancy BMI, and recommended GWG for each woman in both groups. All participants received a meal plan with a list of equivalents based on the traditional or DASH diet, which was explained in detail and revised at each appointment with the registered dietitian, with reinforcement of the nutritional orientations for both diets until the last visit [28].

Adherence to the diets was assessed using a 24-h dietary recall and by applying a tool with four evaluation items, which was scored from 0 to 4 points according to (1) quantity of food consumed—portions; (2) food groups consumed—variety; (3) consumed meals—number and time; and (4) gestational weight gain—adequate when no more than 20% less or above the recommended amount [29]. The adherence score was stratified into low-to-moderate adherence (<2 points) and high adherence (≥2 points). For the present analyses, we considered the adherence score obtained at the visit closest to childbirth to reflect the longest possible time of exposure to the intervention.

To improve adherence, participants in the traditional diet group received a bottle of extra virgin olive oil (500 mL) at the first visit, a can of powdered semi-skimmed milk (300 mg), and a pack of oats (250 mg) at each subsequent visit, while the participants in the DASH diet group received a bottle of extra virgin olive oil (500 mL) at the first visit, a can of powdered skimmed milk (280 mg), and a pack of nuts (150 mg) and seeds (200 mg) at each subsequent visit.

### 2.4. Outcome

The main outcome was excessive GWG (kg). The recommended GWG was defined according to the Institute of Medicine guidelines [6,7] considering the pre-pregnancy BMI: underweight, 12.5 to 18 kg; normal weight, 11.5 to 16 kg; overweight, 7 to 11.5 kg; and obesity, 5 to 9 kg. Excessive GWG was the total weight gain of pregnancy exceeding the maximum amount recommended by IOM [6,7] according to pre-pregnancy BMI: GWG > 18 kg, >16 kg, >11.5 kg, and >9 kg for underweight, normal weight, overweight, and obese women, respectively. The time until excessive GWG was estimated by linear interpolation, assuming a linearly increasing weight gain between different measurements.

To calculate the pre-pregnancy BMI, height was measured during the first prenatal visit, and weight was measured at each prenatal visit by nursing technicians and registered in the medical record. Pre-pregnancy weight was the self-reported weight of a woman near conception [30]. The pre-pregnancy BMI was calculated as (pre-pregnancy weight/height^2^) and was classified as underweight (BMI < 18.5 kg/m^2^), normal weight (BMI 18.5–24.9 kg/m^2^), overweight (BMI 25.0–29.9 kg/m^2^), or obesity (BMI ≥ 30.0 kg/m^2^) [31].

The last pregnancy weight was measured on admission for childbirth. GWG was calculated as (weight at admission for childbirth–pre-pregnancy weight), representing the total weight gain during pregnancy.

### 2.5. Genotyping

We collected saliva samples from each pregnant woman participating in the study, and genomic DNA was isolated from buccal epithelial cells using the Aidar and Line (2007) protocol [32]. FTO (rs9939609 and rs17817449) and ADRB2 (rs1042713 and rs1042714) polymorphisms were genotyped by real-time PCR using TaqMan^®^ assays (Thermo Fisher Scientific, Carlsbad, CA, USA). Reactions were performed in 10-μL volumes containing DNA (2 μL), Universal Master Mix (5 μL), TaqMan Genotyping Assay specific for each polymorphism (0.25 μL), and MiliQ (2.75 μL). Amplification was carried out in a StepOne^®^ Plus Real-Time PCR System (ThermoFisher) according to the manufacturer’s recommendations for the number of cycles and temperatures. Negative and positive controls were included in the plate.

### 2.6. Co-Variates

Data, such as age (years), DM type (1 or 2), education level (elementary, middle, or high school), marital status (married/single), employment (yes/no), per capita income (total family income divided by the number of persons living in the same house, in USD), housing conditions (adequate when all had regular garbage collection, tap water, and sewerage system), pre-existing chronic diseases (hypothyroidism or chronic hypertension), and parity (number of previous childbirths) were obtained from medical records and were complemented in a personal interview with the researchers using structured questionnaires. Physical activity was assessed at baseline using the short form of the International Physical Activity Questionnaire (active, irregularly active, and sedentary) [33].

Skin color (white/black/brown/yellow) and years living with DM were self-reported. Energy intake (kcal) at baseline was obtained using a 24-h dietary recall from which the reported portions of food were converted into grams to quantify the energy content using food composition tables [34,35]. Gestational age (weeks) was calculated using the first ultrasonography performed in prenatal care, which was obtained at the time of inclusion in the study (all <28 weeks of pregnancy).

### 2.7. Statistical Analyses

Data are presented as medians and interquartile ranges (IQR) for numeric variables, and absolute (*n*) and relative frequencies (%) for categorical variables. The normality of the continuous variables was assessed using histograms, kurtosis, and asymmetry measures. Mann–Whitney U and Kruskal–Wallis tests were used to compare continuous numerical variables, and chi-square or Fisher’s exact tests were used for categorical variables. Genotype and allele frequencies of each variant were determined by direct counting, and deviations from Hardy–Weinberg equilibrium (HWE) were evaluated using chi-square tests.

Paired linkage disequilibrium (LD) patterns were determined for each gene using r^2^ statistics (r^2^ cutoff ≥ 0.8). Haplotype frequencies or allelic phase determination were estimated by expectation maximization (EM algorithm), and estimation uncertainty was included in the statistical models applied for association analyses in the form of weights. The homozygous/heterozygous genotypes and lower frequency alleles (minor allele frequency, MAF) in our population, or those containing them, were compared with the higher frequency alleles or genotypes containing higher frequency alleles (reference). Haplotype analyses used the most common haplotype in our population as a reference.

The incidences of excessive GWG were analyzed based on the events and years of persons at risk based on the follow-up time from the most likely date of conception to the most probable date of the outcome. Incidences and 95% confidence intervals (95% CIs) were estimated according to asymptotic standard errors calculated from a gamma distribution. Pregnant women who did not present with the outcome were considered from the most likely date of conception and censored on the day of delivery.

The results of the time-to-event analyses were presented in the form of hazard ratios (HRs) with 95% CIs, and the risks of progression to the events described above were estimated using Cox proportional hazard models. The assumption of risk proportionality was tested using correlation analyses and χ^2^ tests based on Schoenfeld scaled residuals and transformed survival times. The effects of the genetic characteristics of interest were corrected for phenotypic characteristics with at least one suggested association (*p*-value ≤ 0.1) with the outcome of interest and the marginal effects presented in the form of aHR. Pre-gestational BMI was not included in the adjusted model because of its potential mediating effect between genotype and outcome. Each polymorphism was evaluated using the additive, dominant, and recessive models.

Statistical analyses were performed using R software (Version 4.1.1) and its “genetics” and “survival” packages. Power analysis and sample size estimates were performed using the R code available on the Power and Sample Size platform (http://powerandsamplesize.com/Calculators/Test-Time-To-Event-Data/Cox-PH-2-Sided-Equality accessed on 18 January 2022). Considering the overall prevalence of the event of 50%, the frequency of minor allele carriers of 35%, a mean hazard ratio of 2, and alpha = 0.05, the minimum sample size for Cox proportional models estimated for power (1-eta) of 0.8 was 144. Nonetheless, we had a limited sample size (*n* = 70) that reached 56.35% statistical power for this analysis.

## 3. Results

**Figure 1:**
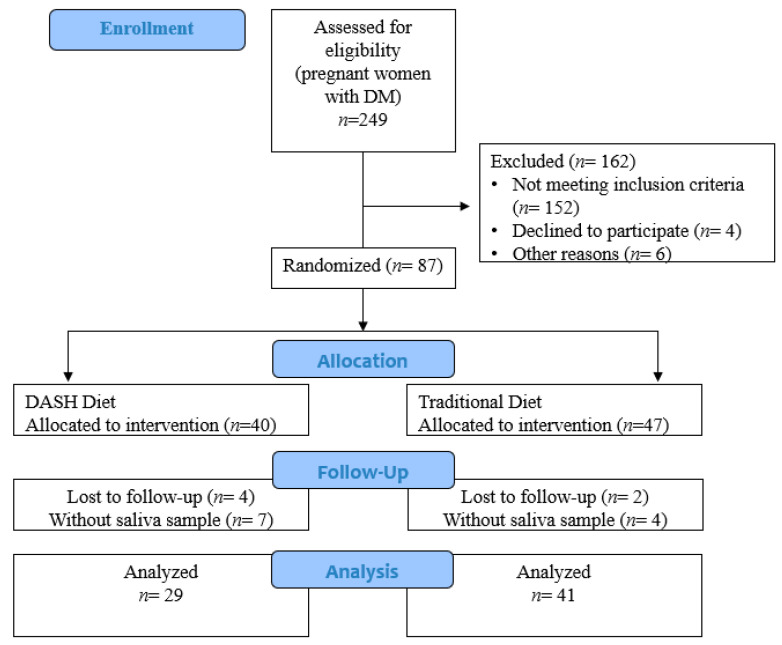
Flowchart of the study (Rio de Janeiro/Brazil, 2016–2020).

Of the 249 pregnant women with pregestational DM assessed for eligibility, 87 were included in the DASDIA clinical trial, and 70 were included in the present study because there were sufficient data for the analyses: 41 in the traditional diet group and 29 in the DASH diet group (Figure 1).

The median age was 32 years (IQR 25.7–36.0), and the gestational age at randomization was 15 weeks (IQR 11.1–20.1). DM type 1 was 51.4% (*n* = 36) of the cases. The distribution of the variables was homogeneous among the diet groups (Table 2).

The genotypic frequencies of rs9939609–FTO were TT 40%, AT 48.6%, and AA 11.4%, while for rs17817449–FTO, the values were TT 45.7%, GT 44.3%, and GG 10%, without differences among diet groups (*p* = 0.48 and *p* = 0.73, respectively; Table 3). The MAFs for rs9939609 (A) were 35.7%, while it was 32.1% for rs17817449 (G).

The genotypic frequencies of rs1042713–ADRB2 were GG 35.7%, AG 52.9%, and AA 11.4%, while for rs1042714–ADRB2, the values were CC 50%, CG 44.3%, and GG 5.7%, without differences among diet groups (*p* = 0.35 and *p* = 0.28, respectively; Table 3). The MAFs for rs1042713 (A) were 37.9% and 27.9% for rs1042714 (G). The genotypes of all evaluated polymorphisms were in HWE (*p* > 0.05).

The median time of intervention was 22.50 weeks (IQR 15.50–26.04). Most pregnant women attended six scheduled appointments (*n* = 38, 54.3%) or at least five of them (*n* = 16, 22.9%). Almost 40% of the participants had the highest adherence scores in both groups (39.5% in the traditional diet group and 40.7% in the DASH diet group).

In the overall sample (*n* = 70), 28.6% of the women had a normal pre-pregnancy BMI, 35.7% were overweight, and 35.7% were obese. None of the pregnant women were underweight pre-pregnancy. The median GWG was 13.7 kg (IQR 11.5–17.5), 11.8 kg (IQR 7.5–16.4), and 11.0 (IQR 5.9–14.1) for normal-weight, overweight, and obese women, respectively, without differences between diet groups (Supplementary Table S1). We found no statistically significant interaction between diet and genotype on GWG but a marginal effect for the AA genotype of rs9939609–FTO and GG genotype of rs17817449–FTO (*p* = 0.05 and *p* = 0.08, respectively) on higher GWG comparing to another genotypes, only in the traditional diet group (Supplementary Figure S1).

Thirty-seven pregnant women (52.9%) presented with excessive GWG, and the median gestational age of exceeding GWG was 31.6 weeks (IQR 26.6–35.0). Compared to the traditional diet, the DASH diet did not modify the risk of progression to excessive GWG (aHR 1.32, CI 95% 0.62–2.79; *p* = 0.46) in our sample. Instead, the time of living with DM ≥ 8 years (aHR 1.99, CI 95% 1.01–3.93; *p* = 0.04), pre-pregnancy overweight (aHR 3.15, CI 95% 1.23–8.09; *p* = 0.02) or obesity (aHR 2.87, CI 95% 1.11–7.42; *p* = 0.03) status, previous hypothyroidism (aHR 4.37, CI 95% 1.62–11.77; *p* = 0.00), and yellow color of the skin (aHR 74.40; CI 95% 4.25–1302.72; *p* = 0.00, not shown in the table) were risk factors for earlier GWG. In contrast, age ≥ 32 years was a protective factor (aHR 0.41, CI 95% 0.21–0.80; *p* = 0.01) (Table 4).

Adjusting for the main confounders, the A allele carriers (AT/AA) had a higher risk of earlier exceeding GWG (aHR 2.55; CI 95% 1.14–5.69; *p* = 0.02) than the rs9939609 TT genotype in the FTO gene, which was also found in the comparison of AT vs. TT genotypes (aHR 2.44; CI 95% 1.03–5.78; *p* = 0.04) (Table 5).

The A allele carriers for rs1042713 in the ADRB2 gene had a higher risk of earlier exceeding GWG than the GG genotype (aHR 2.37; CI 95% 1.01–5.52; *p* = 0.04), being almost four times higher for the AA carriers (aHR 3.91; CI 95% 1.12–13.70; *p* = 0.03). We found that the genotypes for rs17817449 (FTO) and rs1042714 (ADRB2) had no effect on the risk of excess GWG in our sample (Table 6).

Although rs17817449 alone was not associated with the outcome, in the haplotype analysis of rs9939609:rs17817449 (TT/AG/AT), we found a higher risk for earlier excessive GWG among AG carriers: the A allele for rs9939609 and the G allele for rs17817449 (aHR 1.79; CI 95% 1.04–3.06; *p* = 0.02). We found no association between haplotype analysis of the ADRB2 gene in our sample (Table 7).

## 4. Discussion

In a sample of 70 Brazilian pregnant women with pregestational DM, we found that the A allele carriers for rs9939609 (FTO gene) and rs1042713 (ADRB2 gene) had more than twice the risk of earlier exceeding GWG compared to TT and GG genotypes, respectively. The haplotype rs9939609:rs17817449 (AG) was also a risk factor, increasing 1.8 times in terms of the progression to excessive GWG. Time of living with DM of ≥8 years, pre-pregnancy overweight or obesity, and previous hypothyroidism were risk factors for earlier excessive GWG. However, age ≥ 32 years old was a protective factor. We found no effect of the DASH diet on the risk for progression to excessive GWG, but our results of non-association need to be interpreted with caution because of our limited statistical power.

The allele frequencies in our sample were close to the frequencies in global databases (www.ncbi.nlm.nih.gov/snp (accessed on 18 January 2022)). Common polymorphisms (>5% allele frequency) are expected to be shared across different geographical regions and populations [36]. However, the Brazilian population is highly admixed and underrepresented in genomic studies as a potential source of new phenotype-associated genetic variants [37].

The A allele for the rs9939609 polymorphism in the FTO gene was previously associated with higher BMI before and after pregnancy [38,39] but not with excessive GWG [15] in Brazilian women. Studies from the USA [12,13] and Spain [14] found a higher risk of having higher GWG among A-allele carriers, contradicting the results from Turkey [40] and Mexico [41], both without association. The other evaluated polymorphisms (FTO rs17817449, ADRB2 rs1042713, and rs1042714) have not been previously investigated for GWG. The haplotype rs9939609:rs17817449 (AT) was found to increase the risk of obesity in the Brazilian adult population [11], but we identified the role of AG in the risk of progression to GWG.

The effect of the DASH diet on weight gain during pregnancy is controversial. Van Horn et al. (2018) noted that overweight and obese pregnant women (*n* = 280, EUA) gained less weight and had less excessive GWG using the DASH diet than by using a control diet [42], whereas Fulay et al. (2018) reported that high adherence to the DASH diet was associated with more weight gain during pregnancy in obese women (*n* = 1760, EUA) [43].

Genetics may partially explain the interindividual variation in body weight in response to nutritional intervention [44]; thus, it was hypothesized that the FTO and ADRB2 polymorphisms could modify the effect of diet on GWG. We found a marginal association of the FTO polymorphisms on the GWG according to the type of diet: women with the AA genotype for rs9939609 and women with the GG genotype for rs17817449 had higher GWG in the traditional diet group. This result could represent some benefit of the DASH diet on limiting GWG for women with these genotypes, but it was not confirmed in the analysis adjusted for the main confounders, as we found no effect of diet on the risk for progression to excessive GWG in our sample.

Martins et al. (2018) reported that the A allele for rs9939609 was associated with an increase in the total energy intake and increase in the percentage of energy from ultra-processed foods during pregnancy in a cohort of Brazilian women [45]. In our study, we calculated the individualized meal plan for all participants in both groups, considering the appropriate GWG, and we found similar high adherence to diet in the groups (40%). Indeed, the evaluation of dietary intake in details deserves further analyses to clarify if the genetic polymorphisms affect the GWG by dietary characteristics other than the dietary pattern (traditional or DASH), such as the level of food processing, for example.

Pregestational overweight and obesity were risk factors for progression to excessive GWG in our sample. This result agrees with the study by Brandão et al. (2021), which involved a large cohort of healthy Brazilian women and found that excessive GWG was observed in 30.1%, 30.7%, 56.4%, and 46.2% of underweight, normal weight, overweight, and obese women, respectively [2]. According to the IOM guidelines, GWG recommendations decrease when BMI increases. Thus, obese women should gain less weight than overweight and normal weight women, but the guidelines do not provide specific recommendations for women with pregestational DM [6].

In this context, Siegel et al. (2015) found no difference between BMI classes to gain less, within, or above the IOM recommendations in a sample of women with pregestational DM but noticed a higher risk for macrosomia (aOR 4.02; CI 95% 1.16–13.9) and large-for-gestational-age infants (aOR 3.08; CI 95% 1.13–8.39) in women who gained excessive weight compared to women who gained weight within the recommended amounts, even after adjusting for pregestational BMI [46]. The results from the study by Egan et al. (2014) were similar, reporting that excessive GWG in women with pregestational DM was a risk factor for macrosomia (aOR 3.58; CI 95% 1.77–7.24) and large-for-gestational-age infants (aOR 3.97; CI 95% 1.85–8.53), but they found more women with overweight or obesity presenting with excessive GWG than non-excessive GWG (44% vs. 27% for overweight and 37% vs. 25% for obesity; *p* < 0.01) [3].

We found that the number of years living with DM was a risk factor for progression to excessive GWG, but older age (≥32 years) was a protective factor, which appears controversial. The studies by Egan (2014) and Siegel (2015) did not find any association between age and excessive GWG [3,46], but in a cohort of 8184 healthy Brazilian women, the GWG decreased as the age increased [2].

A longer time of living with DM is expected in women with type 1 DM, who usually have lower pregestational BMI compared to those with type 2 DM [47]. Given our results for the effect of pregestational BMI on excessive GWG, it seems contradictory. However, the type of DM was not a risk factor for earlier excessive GWG in our sample. Therefore, we suggest that longer years of living with DM may impact the metabolic and behavioral factors influencing GWG not covered in the present study.

Only one participant had yellow skin. This woman was overweight before pregnancy and had the highest GWG in our sample (28.2 kg). Therefore, even though we had found the yellow color of the skin to be a risk factor for progression to excessive GWG, we considered that it cannot be properly interpreted or discussed, as it was an isolated case with a very wide confidence interval. Three women who had excessive GWG in our sample reported the color of the skin as unknown.

A meta-analysis comparing the prevalence of excessive GWG among racial/ethnic groups found that White women were more likely to exceed the IOM guidelines than their Asian and Hispanic counterparts, but White and Black women had a similar prevalence of excessive GWG [48]. Differences in the GWG regarding the color of the skin are often related to socioeconomic discrepancies [48]. We found a marginal effect of inadequate housing conditions on the risk of earlier excessive GWG, but it was not statistically significant.

Of the nine women with hypothyroidism included in our sample, eight had excessive GWG, with a risk more than four times higher than that of women without hypothyroidism (aHR 4.37, CI 95% 1.62–11.77; *p* = 0.00). Collares et al. (2017) found that higher maternal TSH and lower free T4 levels in early pregnancy were associated with a higher GWG [49]. Hypothyroidism is a common endocrine disorder that occurs during pregnancy [50]. In our sample, all women diagnosed with hypothyroidism were treated with oral repositioning of the T4 hormone and had adequate hormone levels at the time of inclusion in the study; however, monitoring adherence to treatment and hormone levels during pregnancy was outside the scope of this study.

Identifying risk factors for earlier exceeding GWG and implementing a dietary intervention to mitigate it may help decrease the related adverse outcomes. The sooner the excess weight is present, the more that the metabolic effects can harm the mother and fetus [51]. Of particular interest for pregnant women with DM is that an increase in maternal fat mass during early pregnancy can increase insulin resistance and thus worsen glycemic control [52]. As excess GWG does influence offspring obesity over the short- and long-term [53], faster fist and second trimester GWG but not third trimester was associated with higher mid-childhood adiposity in a cohort of 979 mother–child pairs from whom children were evaluated between 6.6 and 10.9 years of age [54].

Our study is novel in terms of several relevant characteristics. First, we investigated candidate genetic risk factors for progression to excessive GWG, and we did not find previous studies with this objective. Second, we investigated the influence of obesity-related polymorphisms in Brazilian women with pregestational diabetes since studies enrolling Brazilian pregnant women in this field are scarce and were not designed for women with DM. Additionally, women were administered two distinct types of diets, and we noticed that it had no effect on the risk of progression to excessive GWG in our sample.

However, this study had some limitations. First, we had a limited sample size to include in these analyses because the clinical trial was originally designed to analyze the effect of the two types of diets on perinatal outcomes without making use of the nutrigenetics approach. Given the increasing evidence regarding the effects of genetic characteristics and gene-diet interactions on BMI and obesity predisposition, we considered that it should gain more attention in the field of maternal and child nutrition. Therefore, we believe that this study will contribute to paving this way.

Furthermore, we had to make adaptations to maintain the study when the COVID-19 pandemic began in 2020. We used telemedicine to complete the follow-up of six women (8.6% of the sample) who were already enrolled in the study at the beginning of the pandemic quarantine. The same study protocol was used for the present visits. Prenatal visits to the physicians were maintained at the local level of the study, maintaining the measurements of weight at each visit. We also asked the participants to send a photograph of the prenatal card containing this information using a popular free smartphone application. Once the pandemic quarantine began, we did not include more participants in the study.

## 5. Conclusions

In this study, we investigated obesity-related polymorphisms in FTO and ADRB2 genes as candidate genetic risk factors for excessive GWG in pregnant women with pregestational DM using traditional or DASH diets.

Regardless the type of diet, the AT carriers of rs9939609 (FTO gene) had more than twice the risk of earlier exceeding GWG compared to the TT genotype, and the AA carriers of rs1042713 (ADRB2 gene) had almost four times higher risk than the GG carriers. The frequencies of these genotypes in our study population were 48.6% and 11.4%, respectively.

We found no effect of the genotypes of rs17817449 (FTO gene) and rs1042714 (ADRB2 gene) on the risk of progression to excessive GWG; however, the AG carriers for FTO haplotype rs9939609:rs17817449 had almost twice the risk of earlier exceeding GWG compared to TT carriers. Non-genetic characteristics associated with the risk of progression to GWG were time living with DM ≥ 8 years, pre-pregnancy overweight or obesity, and previous hypothyroidism. In contrast, age ≥ 32 years old was a protective factor.

Identifying women at a higher risk for exceeding GWG earlier may help improve nutritional interventions to mitigate this risk. The next step in advancing personalized nutrition is to understand which diet patterns may protect these women against excessive GWG and to investigate other genes and potential gene-diet-environment interactions with effects on GWG.

## Figures and Tables

**Table 1:** Daily composition of the diets used in the study.

	Traditional Diet	DASH Diet
Saturated fatty acids *	9.7% E	7.2% E
Monounsaturated fatty acids *	8.5% E	9.2% E
Polyunsaturated fatty acids *	2.8% E	5.6% E
Fiber	42 g	55 g
Calcium	1500 mg	2280 mg
Magnesium	315 mg	496 mg
Potassium	4081 mg	4418 mg
Sodium	2400 mg	2400 mg

* Expressed as percentual of daily energy intake (% E). Data are presented for a daily energy intake of 2100 kcal, as an example.

**Table 2:** General characteristics of the participants at baseline (Rio de Janeiro/Brazil, 2016–2020).

	Overall *n* = 70	Trad. Diet *n* = 41	DASH Diet *n* = 29	*p*-Value *
Age (years)	32 (25.7–36.0)	31 (25.0–35.0)	34 (28.0–37.0)	0.28
Gestational age (weeks)	15.0 (11.1–20.1)	14.4 (11.6–21.6)	16.0 (10.1–18.6)	0.66
DM type *n* (%)				
DM1	36 (51.4)	21 (51.2)	15 (51.7)	0.97
DM2	34 (48.6)	20 (48.8)	14 (48.3)	
Years living with DM	8 (2.0–13.5)	6 (1.9–12.5)	9 (2.0–14.5)	0.36
Skin color *n* (%)				
Brown	27 (38.6)	15 (36.6)	12 (41.4)	0.59
White	22 (31.4)	12 (29.3)	10 (34.5)	
Black	16 (22.9)	11 (26.8)	5 (17.2)	
Yellow	1 (1.4)	0 (0)	1 (3.4)	
Unknown	4 (5.7)	3 (7.3)	1 (3.4)	
Marital status *n* (%)				
Married	56 (80.0)	33 (80.5)	23 (79.3)	0.57
Single	12 (17.1)	6 (14.6)	6 (20.7)	
Missing	2 (2.9)	2 (4.9)	0 (0)	
Education level *n* (%)				
Elementary/middle school	46 (65.7)	26 (63.4)	20 (69.0)	0.73
High school	23 (32.9)	14 (34.2)	9 (31.0)	
Missing	1 (1.4)	1 (2.4)	0 (0)	
Employment *n* (%)				
Yes	42 (60.0)	26 (63.4)	16 (55.2)	0.41
No	27 (38.6)	14 (34.1)	13 (44.8)	
Missing	1 (1.4)	1 (2.4)	0 (0)	
*per capita* income (USD ^†^)	151.51 (103.04–227.27)	154.54 (113.33–228.78)	136.36 (91.67–221.04)	0.59
Housing conditions				
Adequate	64 (91.4)	37 (90.2)	27 (93.1)	1.00
Inadequate	3 (4.3)	2 (4.9)	1 (3.5)	
Missing	3 (4.3)	2 (4.9)	1 (3.5)	
Parity *n* (%)	1 (0–1.25)	1 (0–1.5)	1 (0–1.5)	0.92
Preexisting chronic disease *n* (%)				
None	48 (68.4)	31 (75.6)	17 (58.6)	0.06
Hypertension	9 (12.9)	4 (9.8)	5 (17.2)	
Hypothyroidism	8 (11.4)	2 (4.9)	6 (20.7)	
Both	1 (1.4)	0 (0)	1 (3.4)	
Missing	4 (5.7)	4 (9.8)	0 (0)	
Pre-pregnancy BMI (kg/m^2^)	27.85 (24.4–32.3)	27.10 (24.3–31.9)	28.60 (25.7–33.3)	0.16
Pre-pregnany BMI *n* (%)				
Normal weight	20 (28.6)	14 (34.1)	6 (20.7)	0.45
Overweight	25 (35.7)	14 (34.1)	11 (37.9)	
Obesity	25 (35.7)	13 (31.7)	12 (41.4)	
Energy intake (kcal)	1808.3 (1578.7–2228.6)	1823.8 (1528.9–2362.2)	1780.7 (1644.5–1968.8)	0.68
Physical Activity *n* (%)				
Active	30 (42.9)	15 (36.6)	15 (51.7)	0.51
Irregularly active	27 (38.6)	17 (41.5)	10 (34.5)	
Sedentary	7 (10.0)	5 (12.1)	2 (6.9)	
Missing	6 (8.6)	4 (9.8)	2 (6.9)	

Data presented as median (interquartile range) or as absolute and relative frequencies *n* (%). ^†^ Estimated for exchange rate of 1 real (BRL) = USD 5.5. * Mann–Whitney U test or Kruskal–Wallis test to compare medians and chi-square test or Fisher exact test to compare frequencies.

**Table 3:** Genetic background of the participants concerning FTO and ADRB2 polymorphisms (Rio de Janeiro/Brazil, 2016–2020).

	Overall *n* = 70	Traditional Diet *n* = 41	DASH Diet *n* = 29	*p*-Value *
FTO rs9939609 *n* (%)				
T Allele	90 (64.3)			
A Allele	50 (35.7)			
TT	28 (40.0)	17 (41.5)	11 (37.9)	0.48
AT	34 (48.6)	21 (51.2)	13 (44.8)	
AA	8 (11.4)	3 (7.3)	5 (17.2)	
FTO rs17817449 *n* (%)				
T Allele	95 (67.9)			
G Allele	45 (32.1)			
TT	32 (45.7)	19 (46.3)	13 (44.8)	0.73
GT	31 (44.3)	19 (46.3)	12 (41.4)	
GG	7 (10.0)	3 (7.3)	4 (13.8)	
ADRB2 rs1042713 *n* (%)				
G Allele	87 (62.1)			
A Allele	53 (37.9)			
GG	25 (35.7)	12 (29.3)	13 (44.8)	0.35
AG	37 (52.9)	23 (56.1)	14 (48.3)	
AA	8 (11.4)	6 (14.6)	2 (6.9)	
ADRB2 rs1042714 *n* (%)				
C Allele	101 (72.1)			
G Allele	39 (27.9)			
CC	35 (50.0)	20 (48.3)	15 (51.7)	0.28
CG	31 (44.3)	17 (41.5)	14 (48.3)	
GG	4 (5.7)	4 (9.8)	0 (0)	

FTO, fat mass and obesity-associated gene; ADRB2, adrenoceptor beta 2 gene. Data presented as absolute and relative frequencies *n* (%). Genotypes were in Hardy–Weinberg equilibrium. * Chi-square test or Fisher’s exact test to compare frequencies.

**Table 4:** Cox proportional hazard models or time-to-event analyses (from conception to excessive gestational weight gain) of diet groups and general characteristics of the participants (Rio de Janeiro/Brazil, 2016–2020).

Characteristics	Outcome	pY	Crude Incidence/ 100 pY (CI 95%)	HR (CI 95%)	*p*-Value	aHR * (CI 95%)	*p*-Value
Overall	37	44.4	83.29 (58.65–114.81)	-	-	-	-
Diet							
Traditional diet	18	26.0	69.12 (40.97–109.25)	Reference	-	Reference	-
DASH diet	19	18.4	103.36 (62.23–161.41)	1.66 (0.87–3.17)	0.12	1.32 (0.62–2.79)	0.46
Type of DM							
DM1	17	23.2	73.36 (42.73–117.46)	Reference	-	Reference	-
DM2	20	21.2	94.12 (57.49–145.37)	1.39 (0.728–2.657)	0.32	0.92 (0.38–2.22)	0.86
Years living with DM (years)							
<8	18	25.3	71.25 (42.23–112.61)	Reference	-	Reference	-
≥8	19	18.4	103.10 (62.07–161.00)	1.62 (0.85–3.09)	0.14	1.99 (1.01–3.93)	0.04
Age (years)							
<32	21	23.0	91.20 (56.46–139.41)	Reference	-	Reference	-
≥32	16	21.4	74.78 (42.74–121.44	0.80 (0.41–1.53)	0.49	0.41 (0.21–0.80)	0.01
Color of the skin							
Brown	14	17.2	81.17 (44.37–136.18)	Reference	-	Reference	-
White	10	14.0	71.30 (34.19–131.11)	0.838 (0.372–1.888)	0.67	0.681 (0.293–1.586)	0.37
Black	10	10.2	98.29 (47.13–180.76)	1.404 (0.622–3.171)	0.41	1.132 (0.458–2.8)	0.79
Marital Status							
Married	30	36.9	81.30 (54.86–116.07)	Reference	-	Reference	-
Single	7	6.8	102.84 (41.35–211.90)	1.44 (0.63–3.29)	0.38	1.92 (0.79–4.68)	0.15
Employment							
Yes	20	26.9	74.18 (45.31–114.56)	Reference	-	Reference	-
No	17	16.7	101.54 (59.15–162.58)	1.40 (0.74–2.68)	0.30	1.45 (0.76–2.79)	0.26
Housing Conditions							
Adequate	35	41.1	85.10 (59.27–118.35)	Reference	-	Reference	-
Inadequate	2	1.1	183.54 (22.23–663.01)	4.49 (1.04–19.43)	0.04	4.25 (0.84–21.59)	0.08
Pre-pregnancy BMI							
Normal weight	6	13.5	44.43 (16.31–96.71)	Reference	-	Reference	-
Overweight	16	15.6	102.54 (58.61–166.52)	3.15 (1.23–8.09)	0.02	3.15 (1.23–8.09)	0.02
Obesity	15	15.3	97.94 (54.82–161.53)	2.87 (1.11–7.42)	0.03	2.87 (1.11–7.42)	0.03
Chronic disease							
None	24	31.1	70.74 (44.33–107.10)	Reference	-	Reference	-
Chronic hypertension	5	5.6	89.26 (28.98–208.30)	1.53 (0.58–4.04)	0.39	1.33 (0.48–3.70)	0.59
Hypothyroidism	7	4.9	141.02 (56.70–290.56)	2.66 (1.13–6.30)	0.02	4.37 (1.62–11.77)	0.00
Both	1	0.7	141.02 (3.57–785.72)	1.64 (0.22–12.19)	0.63	1.21 (0.15–9.98)	0.86

pY, person-years; CI, confidence interval; DASH, Dietary Approach to stop Hypertension; DM, diabetes mellitus; BMI, body mass index. * Adjusted HR for skin color, previous chronic diseases, and housing conditions.

**Table 5:** Cox proportional hazard models or time-to-event analyses (from conception to excessive gestational weight gain) stratified by the FTO polymorphisms rs9939609 and rs17817449 (Rio de Janeiro/Brazil, 2016–2020).

Genotypes		Outcome	pY	Crude Incidence/100 pY (CI 95%)	HR (CI 95%)	*p*	aHR * (CI 95%)	*p*
Overall		37	44.4	83.29 (58.65–114.31)	-	-	-	-
rs9939609								
Additive Model	TT	12	18.6	64.33 (33.24–112.38)	Reference	-	Reference	-
	AT	19	20.6	92.06 (55.43–143.77)	1.56 (0.76–3.21)	0.23	2.44 (1.03–5.78)	0.04
	AA	6	5.1	116.94 (42.92–254.53)	2.08 (0.78–5.55)	0.14	2.83 (0.93–8.62);	0.07
Dominant Model	TT	12	18.6	64.33 (33.24–112.38)	Reference	-	Reference	-
	AT/AA	25	25.8	97.02 (62.78–143.21)	1.66 (0.83–3.30);	0.15	2.55 (1.14–5.69)	0.02
Recessive Model	AA	6	5.1	116.94 (42.92–254.53)	Reference	-	Reference	-
	AT/TT	31	39.3	78.9 (53.61–111.99)	0.62 (0.26–1.48)	0.28	0.54 (0.20–1.49)	0.24
rs17817449								
Additive Model	TT	15	21.0	71.42 (39.97–117.80)	Reference	-	Reference	-
	GT	17	18.8	90.55 (52.75–144.98)	1.30 (0.65–2.61)	0.46	1.66 (0.75–3.65)	0.21
	GG	5	4.6	107.62 (34.94–251.14)	1.54 (0.56–4.25)	0.40	2.18 (0.65–7.33)	0.21
Dominant Model	TT	15	21.0	71.42 (39.97–117.8)	Reference	-	Reference	-
	GT/GG	22	23.4	93.94 (58.87–142.22)	1.35 (0.70–2.60)	0.37	1.74 (0.82–3.70)	0.15
Recessive Model	GG	5	4.6	107.62 (34.94–251.14)	Reference	-	Reference	-
	GT/TT	32	39.8	80.45 (55.03–113.57)	0.74 (0.29–1.90)	0.53	0.60 (0.20–1.83)	0.37

pY, person-years; CI, confidence interval; FTO, fat mass and obesity-associated gene. * Adjusted HR for skin color, previous chronic diseases, and housing conditions.

**Table 6:** Cox proportional hazard models or time-to-event analyses (from conception to excessive gestational weight gain) stratified by the ADRB2 polymorphisms rs1042713 and rs1042714 (Rio de Janeiro/Brazil, 2016–2020).

Genotypes		Outcome	pY	Crude Incidence/100 pY (CI 95%)	HR (CI 95%)	*p*	aHR * (CI 95%)	*p*
Overall		37	44.4	83.29 (58.65–114.31)	-	-	-	-
rs1042713								
Additive Model	GG	10	16.3	61.44 (29.46–112.99)	Reference	-	Reference	-
	AG	22	23.6	93.36 (58.51–141.35)	1.72 (0.81–3.64)	0.16	2.14 (0.89–5.14)	0.09
	AA	5	4.6	109.16 (35.44–254.74)	2.05 (0.70–6.02)	0.19	3.91 (1.12–13.70)	0.03
Dominant Model	GG	10		61.44 (29.46–112.99)	Reference	-	Reference	-
	AG/AA	27		95.93 (63.22–139.57)	1.772 (0.856–3.667)	0.12	2.37 (1.01–5.52)	0.04
Recessive Model	AA	5	4.6	109.16 (35.44–254.74)	Reference	-	Reference	-
	AG/GG	32	39.8	80.32 (54.94–113.38)	0.69 (0.27–1.77)	0.44	0.41 (0.14–1.21)	0.11
rs1042714								
Additive Model	CC	20	21.2	94.11 (57.49–145.35)	Reference	-	Reference	-
	CG	15	20.4	73.41 (41.09–121.08)	0.71 (0.36–1.39)	0.32	0.78 (0.37–1.63)	0.51
	GG	2	2.7	73.05 (8.85–263.88)	0.65 (0.15–2.80)	0.57	0.29 (0.04–1.91)	0.20
Dominant Model	CC	20	21.2	94.11 (57.49–145.35)	Reference	-	Reference	-
	CG/GG	17	23.2	73.37 (42.74–117.47)	0.70 (0.37–1.34)	0.29	0.70 (0.34–1.44)	0.33
Recessive Model	GG	2	2.7	73.05 (8.85–263.88)	Reference	-	Reference	-
	CG/CC	35	41.7	83.96 (58.48–116.77)	1.30 (0.31–5.40)	0.72	3.20 (0.48–21.53)	0.23

pY, person-years; CI, confidence interval; ADRB2, adrenoceptor beta 2 gene. * Adjusted HR for skin color, previous chronic diseases, and housing conditions.

**Table 7:** Cox proportional hazard models or time-to-event analyses (from conception to excessive gestational weight gain) of the haplotypes ADRB2 rs1042713:rs1042714 and FTO rs9939609:rs17817449 (Rio de Janeiro/Brazil, 2016–2020).

Haplotypes	Outcome	pY	Crude Incidence/ 100 pY (CI 95%)	HR (CI 95%)	*p*-Value	aHR * (CI 95%)	*p*-Value
ADRB2 rs1042713:rs1042714							
AC	32	32.7	97.78 (66.88–138.04)	Reference	-	Reference	-
GC	23	30.2	76.13 (48.26–114.24)	0.74 (0.43–1.26)	0.26	0.63 (0.36–1.12)	0.12
GG	19	25.9	73.33 (44.15–114.52)	0.67 (0.38–1.19)	0.17	0.59 (0.32–1.09)	0.09
FTO rs9939609:rs17817449							
TT	43	57.2	75.17 (54.40–101.26)	Reference	-	Reference	-
AG	27	27.3	98.81 (65.12–143.77)	1.37 (0.85–2.22)	0.18	1.79 (1.04–3.06)	0.02
AT	4	3.6	111.87 (30.48–286.42)	2.03 (0.73–5.67)	0.26	1.40 (0.46–4.28)	0.49

pY, person-years; CI, confidence interval; FTO, fat mass and obesity-associated gene; ADRB2, adrenoceptor beta 2 gene. * Adjusted HR for skin color, previous chronic diseases, and housing conditions.

## Supplementary Materials

The following supporting information can be downloaded at: https://www.mdpi.com/article/10.3390/nu14051050/s1, Table S1: Comparison of gestational weight gain (kg) between diet groups according to pregestational BMI (Rio de Janeiro/Brazil, 2016–2020); Figure S1: Gestational weight gain according to genotypes and dietary patterns.
