# Supplementary material for: FTO and ADRB2 Genetic Polymorphisms Are Risk Factors for Earlier Excessive Gestational Weight Gain in Pregnant Women with Pregestational Diabetes Mellitus: Results of a Randomized Nutrigenetic Trial
Source: Nutrients. 2022 Mar 1;14(5):1050. doi: 10.3390/nu14051050 (PMC8912276; doi:10.3390/nu14051050)
Supplement: Supplementary file 1 [file nutrients-14-01050-s001.zip › nutrients-1580872-supplementary.pdf]

Supplementary Table S1–Comparison of gestational weight gain (kg) between diet groups according to pregestational BMI (Rio de Janeiro/Brazil, 2016–2020).

	**Overall** n **= 70**	**Traditional Diet** n **= 41**	**DASH Diet** n **= 29**	***p*-Value***
**Normal weight** ǀ BMI 18.5–24.9 kg/m² ǀ Recommended GWG: 11.5 to 16 kg				
	13.7 (11.5–17.5)	13.7 (11.6–15.6)	15.5 (10.0–19.9)	0.57
**Overweight** ǀ BMI 25.0 a 29.9 kg/m² ǀ Recommended GWG: 7 to 11.5 kg				
	11.8 (7.5–16.4)	11.0 (7.6–16.3)	12.9 (7.0–16.8)	0.93
**Obesity** ǀ BMI ≥ 30.0 kg/m² ǀ Recommended GWG: 5 to 9 kg				
	11.0 (5.9–14.1)	9.0 (5.6–13.6)	11.2 (8.9–14.4)	0.50

BMI, body mass index; GWG, gestational weight gain.
Data presented as median and interquartile range.
* U Mann–Whitney test.

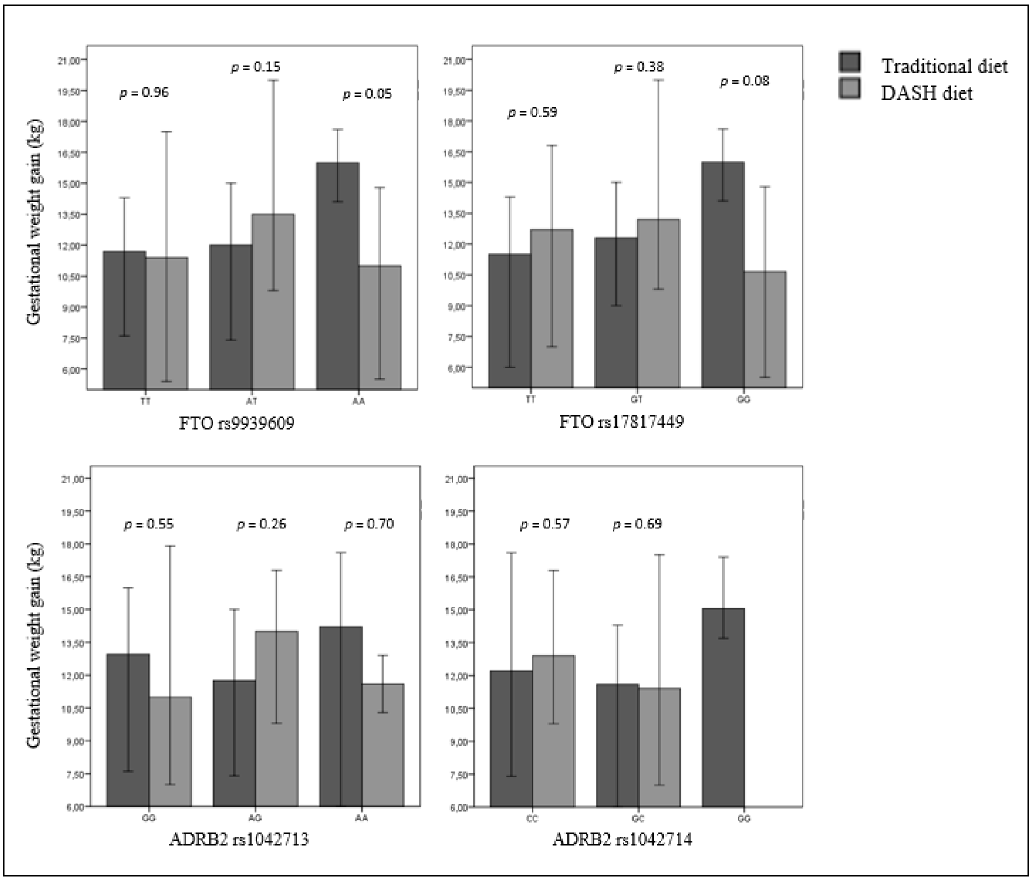

Supplementary Figure S1. Gestational weight gain according to genotypes and dietary patterns. FTO, fat mass and obesity gene; ADRB2, adrenoceptor beta 2 gene; DASH, Dietary Approach to Stop Hypertension. Gestational weight gain (kg) presented as median and error bars 95% CI; *p*-values resulting from Mann–Whitney U test.
